# Sulforaphane prevents doxorubicin-induced oxidative stress and cell death in rat H9c2 cells

**DOI:** 10.3892/ijmm.2015.2199

**Published:** 2015-04-28

**Authors:** BO LI, DO SUNG KIM, RAJ KUMAR YADAV, HYUNG RYONG KIM, HAN JUNG CHAE

**Affiliations:** 1 Department of Pharmacology and Institute of Cardiovascular Research, School of Medicine, Chonbuk National University, Jeonju, Chonbuk 561-180, Republic of Korea; 2 Department of Dental Pharmacology and Wonkwang Biomaterial Implant Research Institute, School of Dentistry, Wonkwang University, Iksan, Chonbuk 570-749, Republic of Korea

**Keywords:** doxorubicin, cardiotoxicity, reactive oxygen species, sulforaphane, NF-E2-related factor-2, heme oxygenase-1

## Abstract

Sulforaphane, a natural isothiocyanate compound found in cruciferous vegetables, has been shown to exert cardioprotective effects during ischemic heart injury. However, the effects of sulforaphane on cardiotoxicity induced by doxorubicin are unknown. Thus, in the present study, H9c2 rat myoblasts were pre-treated with sulforaphane and its effects on cardiotoxicity were then examined. The results revealed that the pre-treatment of H9c2 rat myoblasts with sulforaphane decreased the apoptotic cell number (as shown by trypan blue exclusion assay) and the expression of pro-apoptotic proteins (Bax, caspase-3 and cytochrome *c*; as shown by western blot analysis and immunostaining), as well as the doxorubicin-induced increase in mitochondrial membrane potential (measured by JC-1 assay). Furthermore, sulforaphane increased the mRNA and protein expression of heme oxygenase-1 (HO-1, measured by RT-qPCR), which consequently reduced the levels of reactive oxygen species (ROS, measured using MitoSOX Red reagent) in the mitochondria which were induced by doxorubicin. The cardioprotective effects of sulforaphane were found to be mediated by the activation of the Kelch-like ECH-associated protein 1 (Keap1)/NF-E2-related factor-2 (Nrf2)/antioxidant-responsive element (ARE) pathway, which in turn mediates the induction of HO-1. Taken together, the findings of this study demonstrate that sulforaphane prevents doxorubicin-induced oxidative stress and cell death in H9c2 cells through the induction of HO-1 expression.

## Introduction

Doxorubicin is a chemotherapeutic agent widely used in the treatment of various types cancers, such as lung, liver, breast, ovarian and bladder cancer (1–6). However, cardiotoxicity is a major side-effect of doxorubicin. Reactive oxygen species (ROS) have been reported as one of the major factors responsible for cardiotoxicity. Doxorubicin is reduced by nicotinamide adenine dinucleotide phosphate-oxidase (NADPH oxidase) to a semi-quinone free radical, which in turn interacts with oxygen to form superoxide, hydroxyl and peroxynitrite free radicals (7). Doxorubicin has been reported to induce oxidative stress and mitochondria-mediated apoptosis in cardiomyocytes, leading to their loss and ultimately contributing to progressive heart failure (8). The administration of antioxidants has been shown to protect cardiac cells from oxidative stress induced by doxorubicin (9–11).

Sulforaphane is a naturally occurring isothiocyanate that is highly abundant in certain cruciferous vegetables (12). L-sulforaphane is the biologically active isomer, whereas D,L-sulforaphane is a synthetic racemic analogue of the broccoli constituent, L-sulforaphane (13). Sulforaphane is an antioxidant with cytoprotective effects and anti-carcinogenic properties (14,15) that has been shown to induce the activity of phase II enzymes, such as heme oxygenase-1 (HO-1), quinone reductase, glutathione S-transferase and glutathione reductase (16). Sulforaphane has also been reported to protect the kidneys (17) and brain (18) against ischemic injury through the induction of the activation of transcription factor, NF-E2-related factor-2 (Nrf2)-dependent phase II enzymes. Under basal conditions, the cytosolic regulatory protein, Kelch-like ECH-associated protein 1 (Keap1), binds tightly to Nrf2, retaining it in the cytoplasm (19). Phase II enzyme inducers can disrupt the Keap1/Nrf2 complex, resulting in the release of Nrf2 and its subsequent translocation to the nucleus. The induction of the activation of antioxidant enzymes has been reported to involve transcriptional activation through antioxidant-responsive elements (AREs) (19).

Among the phase II enzymes, HO-1 has attracted significant attention due to its therapeutic effects against neurodegenerative, cardiovascular and hepatic diseases (20–22). Under conditions of oxidative stress, the induction of HO-1 accounts for the majority of heme breakdown, leading to the formation of biliverdin, carbon monoxide (CO) and ferrous iron. In this way, HO-1 mitigates cellular injury by producing molecules with antioxidant and anti-apoptotic effects (20).

The potential cardioprotective effects of sulforaphane have been confirmed by observing reduced ROS production, an increased cell viability (23), and the attenuation of ischemic heart injury through mitochondrial K_ATP_ channels and antioxidant pathways (24). However, the effects of sulforaphane on the cardiotoxicity induced by doxorubicin have not been well defined to date. Therefore, the aim of this study was to determine whether sulforaphane protects cells against doxorubicin-induced cardiac cell death. Specifically, we focused on whether the cardioprotective effects of sulforaphane are related to the activation of the Keap1/Nrf2/ARE pathway and the subsequent induction of HO-1.

## Materials and methods

### Reagents

Antibodies against cleaved caspase-3 (#9661) and Bax (#2772) were purchased from Cell Signaling Technologies (Beverly, MA, USA). Antibodies against cytochrome *c* (sc-13156), Bcl-2 (sc-7382), HO-1 (sc-10789), histone H3 (sc-8654), Nrf2 (sc-722), Keap1 (sc-365626), Hsp60 (sc-13966) and glyceraldehyde 3-phosphate dehydrogenase (GAPDH) (sc-25778) were purchased from Santa Cruz Biotechnology, Inc. (Santa Cruz, CA, USA). Dulbecco’s modified Eagle’s medium (DMEM), fetal bovine serum (FBS), trypsin and other tissue culture reagents were obtained from Invitrogen Life Technologies, Inc. (Carlsbad, CA, USA). Hoechst 33258, 5,5′,6,6′-tetrachloro-1,1′,3,3′-tetraethylbenzimidazolylcarbocyanine iodide (JC-1), dihydrorhodamine 123 (DHR123) and MitoSOX Red reagent were purchased from Molecular Probes (Eugene, OR, USA). Doxorubicin, and L-sulforaphane and D,L-sulforaphane were purchased from Sigma-Aldrich (St. Louis, MO, USA). All other chemicals were from Sigma-Aldrich.

### Cell culture

The H9c2 rat myoblast cell line (KCLB #21446, Korean Cell Line Bank, Seoul, Korea) was grown in DMEM supplemented with 10% FBS and antibiotics (100 *µ*g/ml streptomycin/100 U/ml penicillin mix) in a humidified atmosphere at 37°C with 5% CO_2_.

### Determination of cell viability

The H9c2 cells were passaged and cultured in 24-well plates at 5×10^4^ cells/well for 1 day. The cells were then treated with L-sulforaphane or D,L-sulforaphane for 2 h prior to the addition of doxorubicin. After 24 h, the cells were assessed by trypan blue exclusion assay using a light microscope to determine the percentage of cell death, as previously described (25). The viable cell ratio was calculated as follows: viable cell ratio (%) = (unstained cell number/total cell number) ×100.

### Morphological detection of apoptotic cells

Hoechst 33258, a fluorescent stain used for labeling DNA, was used to identify the apoptotic H9c2 cells, as described in a previous study (26). Following treatment, the cells were washed 3 times with phosphate-buffered saline (PBS) and then fixed for 5 min with ice-cold 100% methanol. After fixation, the cells were stained with Hoechst 33258 (10 *µ*g/ml) for 10 min at room temperature in the dark. The cells were then washed with PBS 3 times and examined under a fluorescence microscope (Olympus, Tokyo, Japan). The apoptotic cells were identified by characteristic nuclei condensation, fragmentation and bright staining, while the nuclei from normal cells demonstrated a normal uniform chromatin pattern.

### Western blot analysis

Western blot analysis was performed as previously described with some modifications (27). The cells treated for the indicated periods of time were harvested by washing twice with ice-cold PBS on ice. For the preparation of whole-cell lysates, the cells were lysed on ice by the addition of RIPA lysis buffer (25 mM Tris-HCl pH 7.4, 150 mM NaCl, 1% sodium deoxycholate, 1% Triton X-100, 5 mM EDTA, 0.1% SDS) plus protease inhibitor cocktail and phosphatase inhibitor cocktail (Roche Diagnostics, Mannheim, Germany) directly onto the cells. The cell lysates were incubated for 30 min on ice, after which they were centrifuged at 10,000 × g for 20 min at 4°C. The supernatants were then collected and used as protein extracts. Protein extracts were added to sample buffer, boiled in a water bath for 5 min and stored at −80°C until use. Protein extracts (30 *µ*g) were run on polyacrylamide gels and transferred onto PVDF membranes for 40 min at 15 V using a semi-dry transfer system (Bio-Rad, Hercules, CA, USA). The membranes were blocked with 5% non-fat dry milk in 0.05% Tween-20/Tris-buffered saline (T-TBS) for 60 min at room temperature. The blots were then probed overnight at 4°C with the relevant primary antibodies, washed and probed again with species-specific secondary antibodies coupled to horseradish peroxidase (Santa Cruz Biotechnology, Inc.). Chemiluminescence reagents (GE Healthcare, Piscataway, NJ, USA) were added for blot detection. Immunoreactive bands were visualized using a LAS-3000 imaging system (Fujifilm, Tokyo, Japan). Band intensities were measured and quantified using ImageJ software as described by Luke Miller (http://lukemiller.org/journal/2007/08/quantifying-western-blots-without.html).

### Detection of cytochrome c and Bax by western blot analysis

To determine the subcellular localization of cytochrome *c* and Bax, the cells were fractionated using digitonin, as previously described (28). Briefly, the cells were suspended in ice-cold plasma membrane permeabilization buffer (200 *µ*g/ml digitonin, 80 mM KCl in PBS). Following a 5-min incubation on ice, the cells were centrifuged at 800 × g for 5 min at 4°C and the soluble extract was taken as the cytosolic fraction. The insoluble pellet was further resuspended in ice-cold cell lysis buffer and incubated on ice for 10 min. Following centrifugation at 10,000 × g for 10 min at 4°C, the resulting supernatant was taken as the membrane-bound organellar fraction enriched with mitochondria. These two fractions were analyzed by western blot analysis using antibodies specific for cytochrome *c*, Bax, Hsp60 and GAPDH.

### Immunostaining for cytochrome c and Bax

Protocols for the immunofluorescence staining for cytochrome *c* and Bax were used as previously described with some modifications (29). The H9c2 cells were grown on coverglass-bottom dishes and treated with the indicated agents. The cells were then fixed with ice-cold methanol and permeabilized with PBST (PBS containing 0.25% Triton X-100). Following a 30-min incubation in blocking buffer (1% BSA in PBST), the cells were incubated with rabbit anti-Bax antibody (1:300) overnight at 4°C. Subsequently, the cells were washed twice and stained with FITC-conjugated goat anti-rabbit secondary antibody (1:300; A24532; Thermo Fisher Scientific, Rockford, IL, USA) for 1 h. The cells were then incubated with mouse anti-cytochrome *c* antibody (1:300) for 1 h and then stained with TRITC-conjugated goat anti-mouse secondary antibody (1:600; ab6786; Abcam, Cambridge, UK) for 1 h. Finally, the cells were mounted using Vectashield mounting medium containing DAPI, and signals were examined under a fluorescence microscope using FITC, TRITC and DAPI channels.

### JC-1 mitochondrial membrane potential (ΔΨm) assay

ΔΨm was determined by flow cytometry using the J-aggregate forming lipophilic cationic probe, JC-1, according to the manufacturer’s instructions (Molecular Probes). JC-1 stains the mitochondria in cells with a high ΔΨm by forming red fluorescence J-aggregates (30), whereas in cells with depolarized mitochondria, JC-1 is present as a green fluorescent monomer. In this way, mitochondrial depolarization can be determined by a decreased ratio of red-to-green fluorescence intensity. The cells were grown in glass-bottom dishes (SPL Life Sciences Co., Ltd., Pochoen, Korea). Following treatment, JC-1 was dissolved in dimethyl sulfoxide (1 mg/ml), diluted to a final concentration of 1 *µ*g/ml in serum-free medium and then added to the cells followed by incubation for 10 min at 37°C; the cells were then washed twice with PBS. Subsequently, the cells were incubated in 1 ml of culture medium and analyzed under a fluorescence microscope (Olympus).

### DHR123

DHR123 is a cell-permeable fluorogenic probe and an indicator of peroxynitrite levels (31,32). Specifically, DHR123 is oxidized by peroxynitrite to cationic rhodamine 123, which localizes in the mitochondria and exhibits green fluorescence (33). Neither nitric oxide, superoxide, nor hydrogen peroxide alone appear to oxidize DHR (34). In order to determine the level of mitochondrial peroxynitrite by fluorescence microscopy, the cells were grown in glass-bottom dishes (SPL Life Sciences Co., Ltd.). Following treatment, DHR123 was dissolved in dimethyl sulfoxide (5 mM) and diluted to 1.25 *µ*M in serum-free medium. The cells were treated with DHR123 for 30 min at 37°C and then washed twice with PBS. Subsequently, the cells were incubated in 1 ml of culture medium and analyzed under a fluorescence microscope (Olympus).

### MitoSOX

MitoSOX Red is a novel fluorogenic dye that serves as an indicator of mitochondrial superoxide levels in live cells (35,36). MitoSOX Red reagent is live-cell permeant and is rapidly and selectively targeted to the mitochondria. Once in the mitochondria, MitoSOX Red reagent is oxidized by superoxide and emits red fluorescence. In order to determine the levels of mitochondrial superoxide by fluorescence microscopy, the cells were grown in glass-bottom dishes (SPL Life Sciences Co., Ltd.). Following treatment, MitoSOX reagent was dissolved in dimethyl sulfoxide (5 mM), diluted to 5 *µ*M in serum-free medium, and was then added to the cells followed by incubation for 10 min at 37°C; the cells were then washed twice with PBS. Subsequently, the cells were incubated in 1 ml of culture medium and analyzed under a fluorescence microscope (Olympus).

### Detection of Nrf2 and Keap1 by western blot analysis

Nuclear and cytoplasmic extracts of H9c2 cells were prepared using NE-PER nuclear and cytoplasmic extraction reagent (Pierce Biotechnology, Rockford, IL, USA) as recommended by the manufacturer. The two fractions were analyzed by western blot analysis with antibodies specific to Nrf2 and Keap1.

### Immunostaining for Nrf2 and Keap1

The H9c2 cells were grown on coverglass-bottom dishes and treated with the indicated agents. The cells were then fixed with ice-cold methanol and permeabilized with PBST (PBS containing 0.25% Triton X-100). Following incubation for 30 min in blocking buffer (1% BSA in PBST), the cells were incubated with rabbit anti-Nrf2 antibody (1:400) overnight at 4°C. The cells were then washed twice and stained with FITC-conjugated goat anti-rabbit secondary antibody (1:400) for 1 h. Subsequently, the cells were incubated with mouse anti-Keap1 antibody (1:100) for 1 h and then stained with TRITC-conjugated goat anti-mouse secondary antibody (1:200) for 1 h. Finally, the cells were mounted using Vectashield mounting medium with DAPI. Signals were examined by fluorescence microscopy using the FITC, TRITC and DAPI channels.

### Reverse transcription-quantitative PCR (RT-qPCR)

Total RNA was extracted from the H9c2 cells using TRIzol reagent (Invitrogen Life Technologies). The ethanol-precipitated RNA fraction (500 ng) was reverse transcribed using the PrimeScript™ RT reagent kit (RR037A; Takara, Shiga, Japan) according to the manufacturer’s protocol. The primers used for PCR were as follows: 5′-AGAGTTTCCGCCTCCAACCA-3′ and 5′-CGGGACTGGGCTAGTTCAGG-3′ for rat HO-1 and 5′-CAGTCAAGGCTGAGAATGG-3′ and 5′-CGACATACT CAGCACCAGC-3′ for rat GAPDH, as described in a previous study (37). Relative gene expression was determined by quantitative (real-time) PCR (qPCR) using an Applied Biosystems 7900HT Fast Real-Time PCR system (Applied Biosystems, Foster City, CA, USA) according to the instructions provided by the manufacturer.

### ARE luciferase activity assay

The cells were transfected with an ARE promoter luciferase reporter plasmid for 24 h and then treated with doxorubicin in the absence or presence of L-sulforaphane or D,L-sulforaphane for various periods of time. Cell lysates were prepared and assayed for luciferase activity using the Luciferase Assay System (Promega, Madison, WI, USA) according to the manufacturer’s instructions. Changes in luciferase activity with respect to the controls (untreated cells) were then calculated.

### Statistical analysis

All data are presented as the means ± SEM. A two-tailed Student’s t-test was applied to examine the statistical significance of differences between groups. Origin 8.0 software (OriginLab Corp., Northampton, MA, USA) was used for statistical calculations. Values of P<0.05 were considered to indicate statistically significant differences.

## Results

### L-sulforaphane and D,L-sulforaphane protect H9c2 myoblasts against doxorubicin-induced cell death

**Figure 1:**
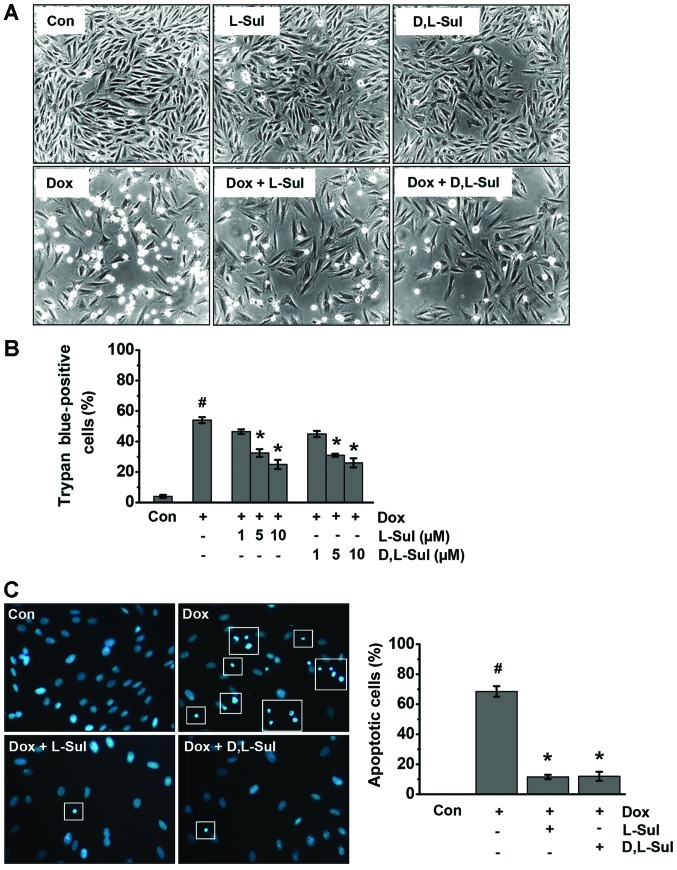
L-sulforaphane (L-Sul) and D,L-sulforaphane (D,L-Sul) protect against doxorubicin (Dox)-induced cell death. (A) H9c2 cells were stimulated with 1 *µ*M Dox or pre-treated with 10 *µ*M L-Sul or D,L-Sul for 2 h and then treated with 1 *µ*M Dox for 24 h. The effects of L-Sul or D,L-Sul on Dox-induced cell death and morphological alterations in the H9c2 cells were observed under a light microscope. (B) Determination of H9c2 cell viability by trypan blue exclusion assay. ^#^P<0.05 vs. controls; ^*^P<0.05 vs. Dox-treated group. (C) Morphological apoptosis was determined by Hoechst 33258 staining under a fluorescence microscope (left panel). Bar graph showing the quantification of apoptotic cells as a percentage of total cells (right panel). White sqaure boxes indicate apoptotic cells. ^#^P<0.05 vs. controls; ^*^P<0.05 vs. Dox-treated group.

The H9c2 cells were exposed to doxorubicin and cell viability was examined after 24 h under a light microscope and by trypan blue exclusion assay. Based on the results obtained by light microscopy, treatment of the H9c2 cells with doxorubicin induced morphological changes, including rounding up and detachment. Treatment with L-sulforaphane and D,L-sulforaphane clearly protected the H9c2 cells against doxorubicin-induced cell death (Fig. 1A). In addition, treatment with either L-sulforaphane or D,L-sulforaphane alone was not toxic to the H9c2 cells. Analysis of trypan blue dye uptake also revealed that both L-sulforaphane and D,L-sulforaphane increased cell viability in a dose-dependent manner (Fig. 1B). The results of the analysis of apoptotic cells using Hoechst 33258 staining are shown on the left panel of Fig. 1C. Following treatment with doxorubicin, the H9c2 cells exhibited numerous brightly condensed and broken fluorescent nuclei. Conversely, the number of apoptotic cells treated with doxorubicin was significantly decreased in the cells pre-treated with L-sulforaphane or D,L-sulforaphane. The results of the quantification of apoptotic cells are shown on the right panel of Fig. 1C.

### L-sulforaphane and D,L-sulforaphane protect H9c2 myoblasts against the doxorubicin-induced translocation of Bax to the mitochondria and the release of cytochrome c

**Figure 2:**
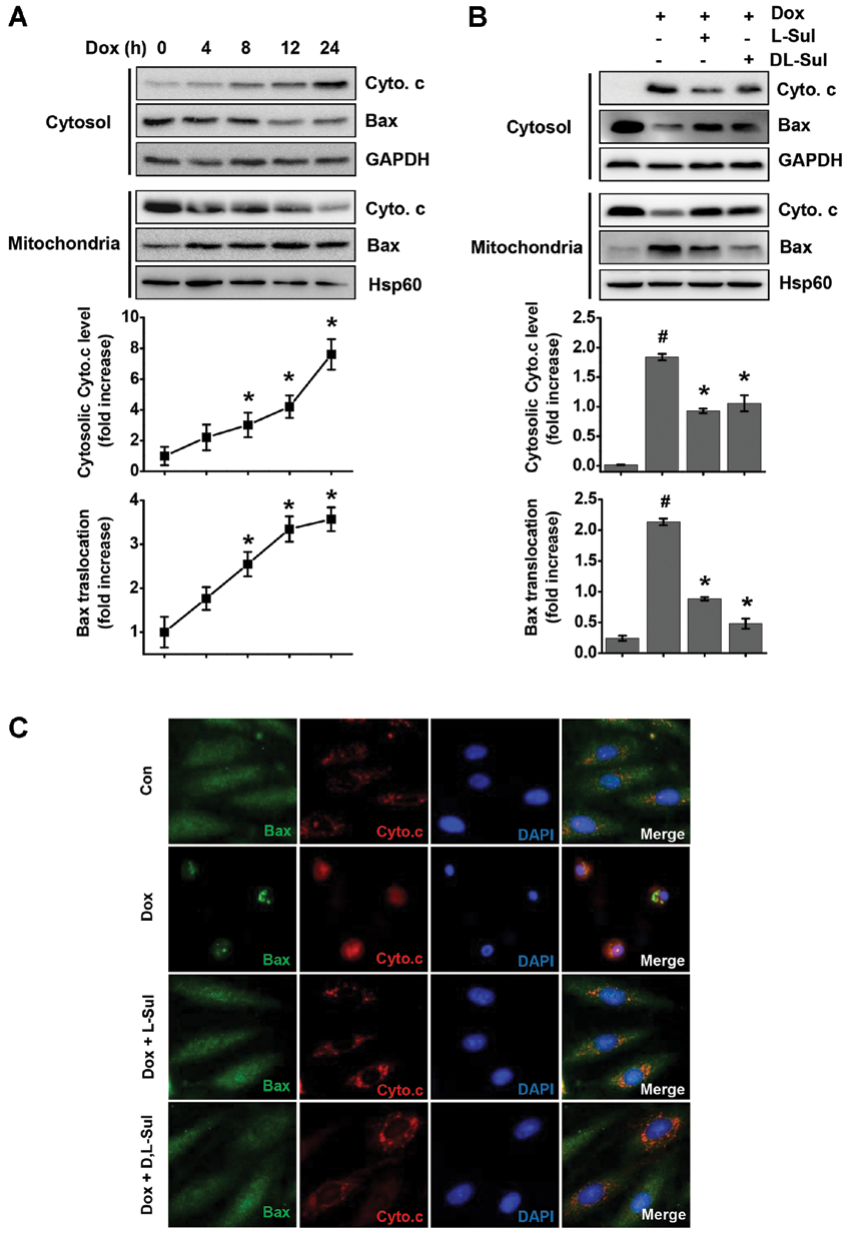
L-sulforaphane (L-Sul) and D,L-sulforaphane (D,L-Sul) prevent the doxorubicin (Dox)-induced release of cytochrome *c* and Bax activation. (A) H9c2 cells were treated with 1 *µ*M Dox for the indicated periods of time. Cells were processed into cytosolic and mitochondrial fractions and subjected to western blot analysis of Bax and cytochrome *c* (upper panel). The lower panel shows the results of densitometric analysis. ^*^P<0.05 vs. controls. (B) H9c2 cells were pre-treated with 10 *µ*M L-Sul or D,L-Sul for 2 h, and then treated with 1 *µ*M Dox for 24 h. Cells were then processed into cytosolic and mitochondrial fractions and subjected to western blot analysis of Bax and cytochrome *c* (upper panel). The lower panel shows the results of densitometric analysis. ^#^P<0.05 vs. controls; ^*^P<0.05 vs. Dox-treated group (C) H9c2 cells were stimulated with 1 *µ*M Dox or pre-treated with 10 *µ*M L-Sul or D,L-Sul for 2 h, and then treated with 1 *µ*M Dox for 24 h. Cells were double-immunostained for Bax and cytochrome *c* and the nuclei were visualized by DAPI staining. Cyto. c, cytochrome *c.*

We then evaluated the effects of L-sulforaphane and D,L-sulforaphane on translocation of Bax to the mitochondria and the subsequent release of cytochrome *c* following treatment with doxorubicin using cellular fractionation and western blot analysis. Kinetic analysis of the appearance of the main signs of apoptosis in the doxorubicin-treated cells revealed the rapid release of mitochondrial cytochrome *c* into the cytosol of H9c2 cells within 4 h of treatment (Fig. 2A). The presence of L-sulforaphane and D,L-sulforaphane prevented the release of cytochrome *c* into the cytosol in comparison to the group treated with doxorubicin alone (Fig. 2B). Similarly, in the cells treated with doxorubicin alone, we observed a time-dependent increase in the translocation of Bax to the mitochondria and a concomitant decrease in cytosolic Bax levels (Fig. 2A). Pre-treatment with L-sulforaphane and D,L-sulforaphane prevented the translocation of Bax into the cytosol compared to the cells treated with doxorubicin alone (Fig. 2B). We also investigated the subcellular distribution of Bax and cytochrome *c* in the H9c2 cells by dual immunofluorescence staining of Bax and cytochrome *c*. The control cells displayed a cytosolic distribution pattern of Bax and a punctate pattern of cytochrome *c* immunostaining (Fig. 2C). During apoptosis induced by doxorubicin, Bax translocated to the mitochondria and displayed a punctate pattern. The Bax-positive cells displayed a diffuse cytosolic pattern of cytochrome *c* staining, as well as a condensed and shrunken nucleus as assessed by Hoechst 33258 staining (Fig. 1C). Consistent with the results from western blot analysis (Fig. 2B), pre-treatment with L-sulforaphane and D,L-sulforaphane prevented the translocation of Bax to the mitochondria and the release of cytochrome *c* (Fig. 2C).

### L-sulforaphane and D,L-sulforaphane prevent the doxorubicin-induced activation of caspase-3 and protect cells against doxorubicin-induced changes in ΔΨm

**Figure 3:**
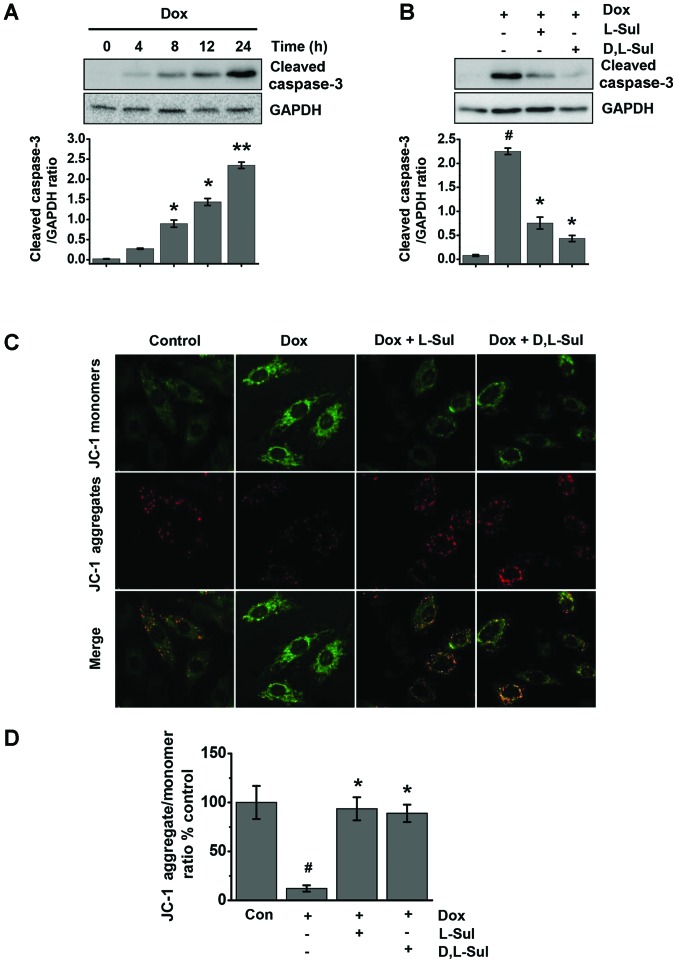
L-sulforaphane (L-Sul) and D,L-sulforaphane (D,L-Sul) prevent the doxorubicin (Dox)-induced activation of caspase-3 and protect against Dox-induced changes in mitochondrial transmembrane potential. (A) H9c2 cells were treated with 1 *µ*M Dox for the indicated periods of time. Cells were lysed and examined for the levels of cleaved caspase-3 by western blot analysis (upper panel). The relative cleaved caspase-3 protein level was normalized to the β-actin level (lower panel). ^*^P<0.05 and ^**^P<0.001 vs. controls. (B) H9c2 cells were pre-treated with 10 *µ*M L-Sul or D,L-Sul for 2 h, and then treated with 1 *µ*M Dox for 24 h. Cells were lysed and examined for the levels of cleaved caspase-3 by western blot analysis (upper panel). Relative cleaved caspase-3 protein levels were normalized to β-actin level (lower panel). ^#^P<0.05 vs. controls; ^*^P<0.05 vs. Dox-treated group. (C) H9c2 cells were stimulated with 1 *µ*M Dox or pre-treated with 10 *µ*M L-Sul or D,L-Sul for 2 h, and then treated with 1 *µ*M Dox for 24 h. Mitochondrial membrane potential was detected by JC-1 fluorescence staining. Specifically, the cells were stained with JC-1 and examined under a fluorescence microscope for the detection of red JC-1 aggregates (590 nm emission) or green JC-1 monomers (527 nm emission). Typical images are shown at ×600 magnification. (D) Quantification of data in (C). ^#^P<0.05 vs. controls; ^*^P<0.05 vs. Dox-treated group.

Doxorubicin was found to induce time-dependent cell apoptosis in the H9c2 cells. Treatment with doxorubicin significantly increased the levels of cleaved caspase-3 at 8 h; these levels increased further in a time-dependent manner (Fig. 3A). As shown in Fig. 3B, both L-sulforaphane and D,L-sulforaphane attenuated the doxorubicin-induced increase in the levels of cleaved caspase-3. ΔΨm is one of the key events during apoptosis (38). Mitochondrial permeability transition has been implicated in the collapse of ΔΨm. To monitor ΔΨm, we used the JC-1 dye and measured the emission ratio at 590 to 527 nm. As shown in Fig. 3C, the control cells exhibited mostly brightly stained mitochondria emitting red fluorescence, whereas the H9c2 cells treated with doxorubicin produced green fluorescence indicative of mitochondrial depolarization and the collapse of ΔΨm. We observed an approximately 80% loss in ΔΨm in the cells treated with doxorubicin alone compared with the control cells (Fig. 3D). Conversely, the cells pre-treated with either L-sulforaphane or D,L-sulforaphane exhibited a significant preservation of red fluorescence compared with the cells treated with doxorubicin alone (Fig. 3C and D).

### L-sulforaphane and D,L-sulforaphane reduce the doxorubicin-induced generation of mitochondrial ROS in H9c2 cells

**Figure 4:**
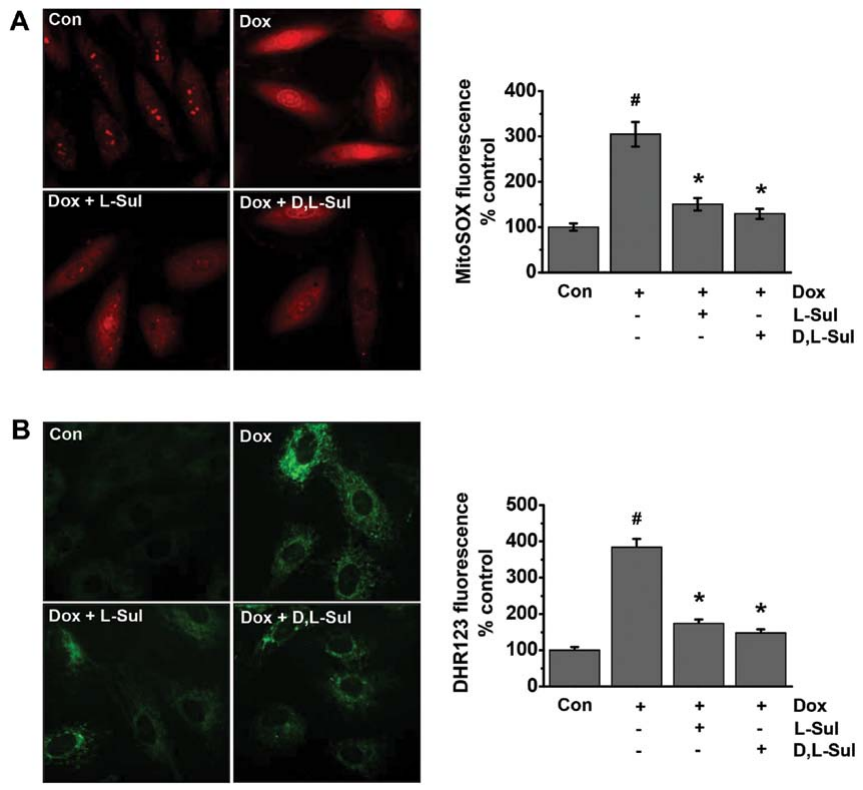
L-sulforaphane (L-Sul) and D,L-sulforaphane (D,L-Sul) reduce the doxorubicin (Dox)-induced generation of mitochondrial reactive oxygen species (ROS) in H9c2 cells. (A) H9c2 cells were treated with 1 *µ*M doxorubicin or pre-treated with 10 *µ*M L-Sul or D,L-Sul for 2 h, and then treated with 1 *µ*M Dox for 24 h. Cells were then analyzed for mitochondrial superoxide generation by fluorescence microscopy using MitoSOX Red. Representative micrographs are shown on the left panel, and quantification plots are shown on the right panel. Results were calculated as a percentage of control in fluorescence intensity compared with the control group. Plots are the means ± SE (n=3). ^#^P<0.05 vs. controls; ^*^P<0.05 vs. Dox-treated group. (B) H9c2 cells were treated with 1 *µ*M Dox or pre-treated with 10 *µ*M L-Sul or D,L-Sul for 2 h, and then treated with 1 *µ*M Dox for 24 h. Cells were then analyzed for mitochondrial peroxynitrite generation by fluorescence microscopy using DHR123. Representative micrographs are shown on the left panel, and quantification plots are shown on the right panel. Results are calculated as a percentage of control in fluorescence intensity compared with the control group. Plots are the means ± SE (n=3). ^#^P<0.05 vs. controls; ^*^P<0.05 vs. Dox-treated group.

ROS are involved in doxorubicin-induced cell apoptosis (39–41). Previous studies have suggested that cardiomyocyte mitochondria are important intracellular targets of ROS during doxorubicin-induced cardiotoxicity. Superoxide and peroxynitrite are the major ROS induced by doxorubicin (42,43). Thus, in the present study, we evaluated whether L-sulforaphane and D,L-sulforaphane influence the generation of doxorubicin-induced ROS. The cells were analyzed for mitochondrial superoxide generation by fluorescence microscopy using the mitochondria-targeted dye, dihydroethidium (MitoSOX Red), as a probe (Fig. 4A). Our results indicated that doxorubicin increased mitochondrial superoxide generation compared to the untreated control cells. Conversely, the doxorubicin-induced MitoSOX fluorescence intensity was attenuated by pre-treatment with L-sulforaphane and D,L-sulforaphane (Fig. 4A). We also investigated the generation of mitochondrial peroxynitrite using the dye, DHR123, as a probe. As shown in Fig. 4B, the doxorubicin-induced DHR123 fluorescence was attenuated by pre-treatment wtih L-sulforaphane and D,L-sulforaphane. Taken together, these results suggest that both L-sulforaphane and D,L-sulforaphane protect H9c2 cells against doxorubicin-induced apoptosis by preventing doxorubicin-induced mitochondrial ROS generation.

### L-sulforaphane and D,L-sulforaphane activate HO-1 through AREs in H9c2 cells

**Figure 5:**
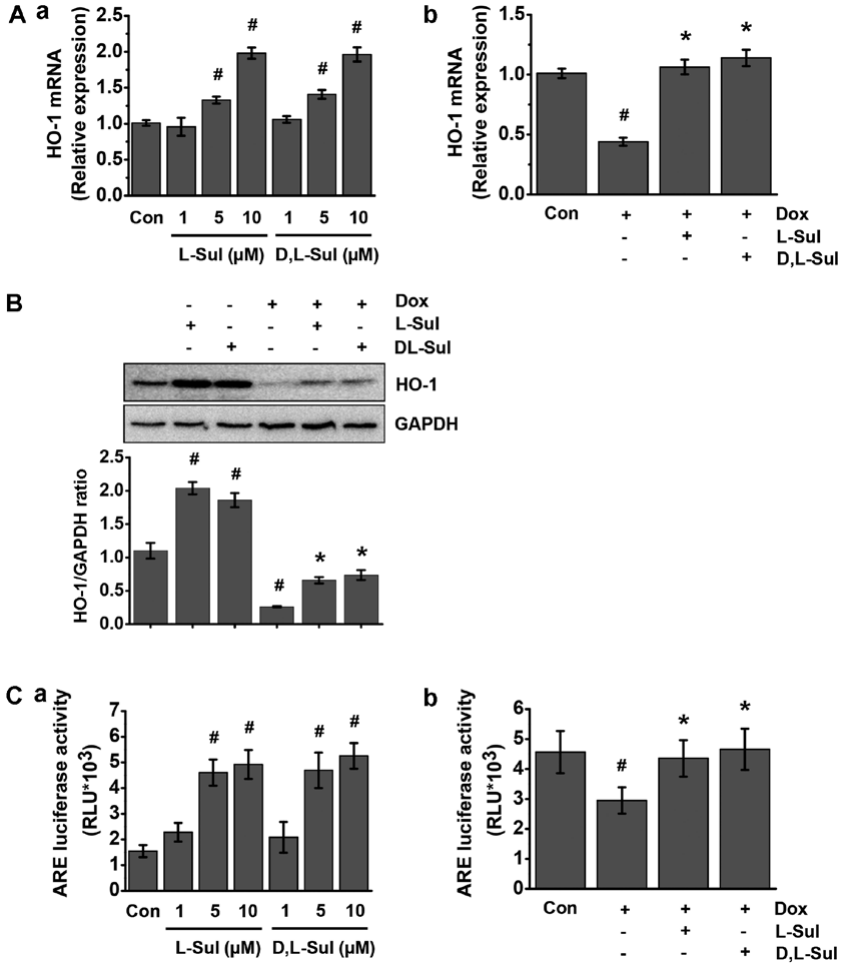
L-sulforaphane (L-Sul) and D,L-sulforaphane (D,L-Sul) activate heme oxygenase-1 (HO-1) through antioxidant-responsive elements (AREs) in H9c2 cells. (A. panel a) H9c2 cells were treated with 10 *µ*M L-Sul or D,L-Sul for 24 h. (Panel b) H9c2 cells were treated with 1 *µ*M doxorubicin (Dox) or pre-treated with 10 *µ*M L-Sul or D,L-Sul for 2 h, and then treated with 1 *µ*M Dox for 24 h. HO-1 mRNA levels were measured by RT-qPCR. ^#^P<0.05 vs. controls; ^*^P<0.05 vs. Dox-treated group. (B) Protein expression of HO-1 and glyceraldehyde 3-phosphate dehydrogenase (GAPDH) was measured by western blot analysis. Densitometric analysis is shown on the lower panel. ^#^P<0.05 vs. controls; ^*^P<0.05 vs. Dox-treated. (C, panel a) H9c2 cells were treated with 10 *µ*M L-Sul or D,L-Sul for 24 h. (Panel b) H9c2 cells were treated with 1 *µ*M Dox or pre-treated with 10 *µ*M L-Sul or D,L-Sul for 2 h, and then treated with 1 *µ*M Dox for 24 h. ARE luciferase activity was measured. ^#^P<0.05 vs. controls; ^*^P<0.05 vs. Dox-treated group.

L-sulforaphane and D,L-sulforaphane protected the H9c2 cells from doxorubicin-induced oxidative insults (Fig. 4). This protective action of sulforaphane may be related to the induction of HO-1, which, along with other phase II enzymes, serves as a defense system against oxidative stress (44,45). Using RT-qPCR, we found that pre-treatment with L-sulforaphane and D,L-sulforaphane induced HO-1 mRNA expression in a dose-dependent manner (Fig. 5A panel a). In addition, pre-treatment with L-sulforaphane and D,L-sulforaphane reversed the decrease in HO-1 mRNA expression observed in the cells treated with doxorubicin alone (Fig. 5A panel b). We then measured HO-1 protein expression levels by western blot analysis. Consistent with our mRNA data, pre-treatment with L-sulforaphane and D,L-sulforaphane induced a significant increase in HO-1 protein expression (Fig. 5B). Furthermore, HO-1 protein expression was higher in the cells pre-treated with L-sulforaphane and D,L-sulforaphane before the addition of doxorubicin compared with cells treated only with doxorubicin. We also found that L-sulforaphane and D,L-sulforaphane stimulated ARE-dependent transcriptional activity in a dose-dependent manner (Fig. 5C panel a). Similarly, pretreatment with L-sulforaphane and D,L-sulforaphane increased ARE-dependent transcriptional activity compared to the cells treated with doxorubicin alone (Fig. 5C panel b). Taken together, these results demonstrate that sulforaphane induces HO-1 mRNA and protein expression by activating AREs.

### Activation of the Keap1/Nrf2 pathway by L-sulforaphane and D,L-sulforaphane in H9c2 cells

**Figure 6:**
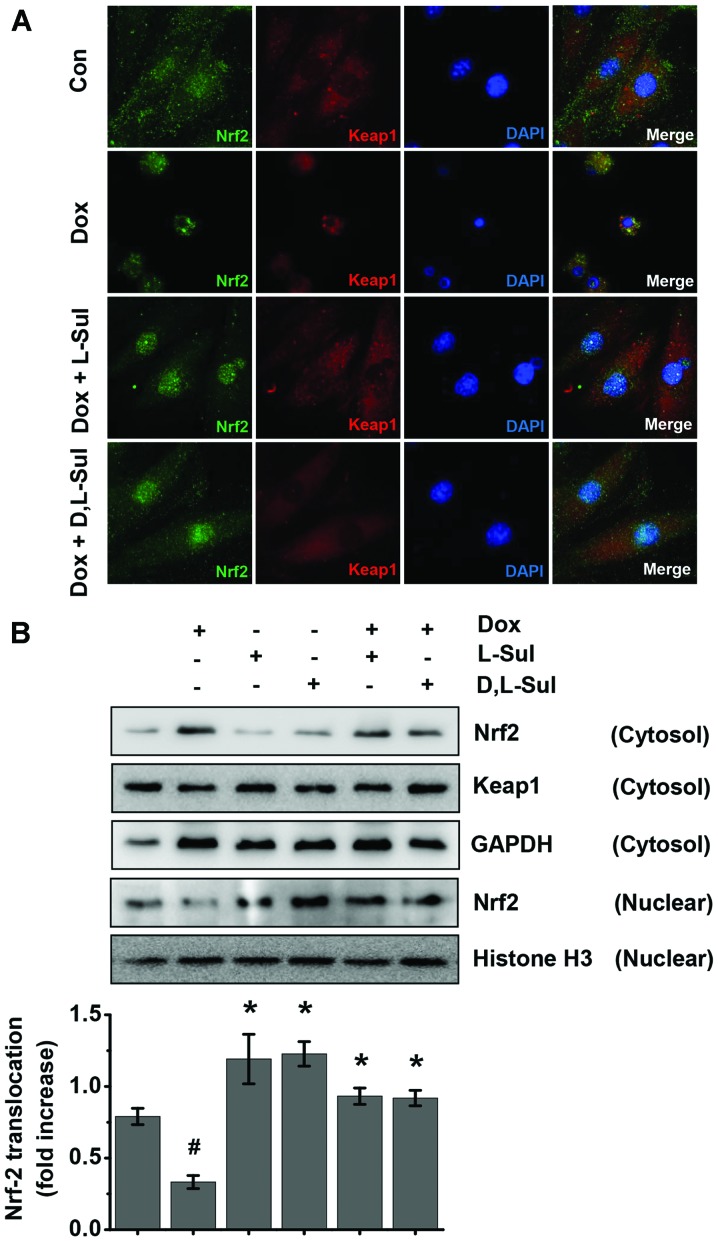
Activation of the Kelch-like ECH-associated protein 1 (Keap1)/NF-E2-related factor-2 (Nrf2) pathway by L-sulforaphane (L-Sul) and D,L-sulforaphane (D,L-Sul) in H9c2 cells. (A) H9c2 cells were treated with 1 *µ*M doxorubicin (Dox) or pre-treated with 10 *µ*M L-Sul or D,L-Sul for 2 h, and then treated with 1 *µ*M Dox for 24 h. Cells were double immunostained for Nrf2 and Keap1 and nuclei were visualized by DAPI staining. (B) H9c2 cells were treated with 1 *µ*M Dox or pre-treated with 10 *µ*M L-Sul or D,L-Sul for 2 h, and then treated with 1 *µ*M Dox for 24 h. In parallel, cells were also treated with 10 *µ*M L-Sul or D,L-Sul alone for 24 h. Nuclear and cytosolic fractions of H9c2 cells were obtained and subjected to western blot analysis using Nrf2 and Keap1 antibodies (top panel). Glyceraldehyde 3-phosphate dehydrogenase (GAPDH) was used as a cytosolic marker, while histone H3 was used to identify nuclear fractions. Densitometric analysis is shown on the lower panel. ^#^P<0.05 vs. controls; ^*^P<0.05 vs. Dox-treated group.

We then investigated the activation status of Nrf2 in the H9c2 cells by assessing the nuclear translocation of Nrf2 by western blot analysis of the cytosolic and nuclear fractions and by immunofluorescence staining of Nrf2 and Keap1 (Fig. 6). Immunofluorescence staining and confocal microscopy revealed that Nrf2 and Keap1 were predominantly localized in the cytoplasm under basal conditions. In the doxorubicin-treated positive cells, Nrf2 was almost completely absent in the nuclear fraction. Conversely, the nuclear Nrf2 content was increased in the presence of L-sulforaphane and D,L-sulforaphane, whereas Keap1 remained localized in the cytoplasm (Fig. 6A). Similarly, western blot analysis revealed that Nrf2 expression induced by L-sulforaphane and D,L-sulforaphane was present at much higher levels in the nucleus than in the cytoplasmic fraction (Fig. 6B). Taken together, these results demonstrate that L-sulforaphane and D,L-sulforaphane activate the Keap1/Nrf2 pathway in H9c2 cells.

## Discussion

Recent evidence suggests a critical role of ROS in doxorubicin-induced cardiotoxicity, leading to left ventricular pathological hypertrophy and ultimately, heart failure (46). Therefore, the induction of the activation endogenous antioxidants and phase II enzymes by dietary means may be a promising cardio-protective strategy. In this study, we found that sulforaphane protected H9c2 cells against cell death induced by doxorubicin. Sulforaphane induced Nrf2-activated HO-1 expression, which consequently reduced ROS levels induced by doxorubicin. In this study, we used H9c2 cells as a model for *in vitro* studies of doxorubicin-induced cardiotoxicity. The H9c2 cell line was originally derived from embryonic rat ventricular tissue (47), which is important to note, as cardiac hypertrophy resulting from hypertension occurs primarily in the ventricular muscle of the heart. Although H9c2 cells have lost their ability to spontaneously contract, they still show many similarities to primary cardiomyocytes (48,49). Indeed, previous studies, using a cell culture approach have investigated doxorubicin-induced cardiac hypertrophy with rat H9c2 ventricular myocardial cells as a model (50,51).

The data from the present study demonstrated that doxorubicin activated apoptotic signaling as indicated by a significant increase in the number of apoptotic cells, the upregulation of pro-apoptotic proteins (Bax, caspase-3 and cytochrome *c*) and an increase in ΔΨm. Excessive oxidative stress by products in the mitochondria likely serves as an upstream trigger of the apoptosis cascade (52). Importantly, oxidative stress-induced apoptosis is a final common pathway for progressive heart failure (53). In this study, pre-treatment of the cells with sulforaphane decreased the number of apoptotic cells, down-regulated the levels of pro-apoptotic proteins (Bax, caspase-3 and cytochrome c) and decreased ΔΨm.

Since ROS act as key mediators in doxorubicin cardiotoxicity models, antioxidant defense mechanisms are necessary to maintain normal cellular function. The Nrf2-phase II enzyme system functions as one of the most important antioxidant defense mechanisms by upregulating antioxidant response element-related detoxification (17). In the present study, we focused on one important Nrf2-target protein, HO-1, which was increased by pre-treatment with sulforaphane during doxorubicin-induced oxidative stress. Specifically, doxorubicin significantly increased mitochondrial ROS levels, whereas pre-treatment with sulforaphane reversed this increase. The reversal effects of sulforaphane towards the mitochondrial ROS levels confirmed its role as an antioxidant in cardiac injury. Although sulforaphane is not a direct antioxidant, it activates the transcription of phase II genes, the products of which provide chemically versatile, often catalytic and prolonged ‘indirect’ antioxidant protection (54). Although several natural or synthetic compounds may be used to prevent doxorubicin-induced cardiotoxicity (11,55–59), one of the major advantages of sulforaphane is that it is already a component of the human diet and is therefore likely to be relatively safe for chronic administration (54). In addition, we observed that L-sulforaphane and D,L-sulforaphane had similar effects. Sulforaphane is one of the promising chemo-preventive phytochemicals (16,60). Sulforaphane is able to increase the efficacy of doxorubicin and induce apoptosis in doxorubicin-resistant p53 mutant cells (60). Sulforaphane has been shown to significantly enhance doxorubicin cytoxicity particularly in A549 lung cancer cells, but not in other cancer cells (61). Additional studies are required to further elucidate the mechanisms involved.

In conclusion, the findings of the present study demonstrated that sulforaphane effectively reduced ROS production and apoptosis induced by doxorubicin in H9c2 cells, and that the protective mechanisms of action of sulforaphane were mediated by preconditioning through the activation of Nrf2 and the subsequent induction of HO-1. Additional studies are warranted to obtain further insight into the clinical modalities through which sulforaphane exerts its beneficial effects on patients who are at risk of heart injury after receiving doxorubicin chemotherapy by improving heart function. Nevertheless, the dietary consumption of sulforaphane-containing cruciferous vegetables, such as broccoli may prove to be useful in patients to prevent doxorubicin-induced cardiotoxicity.
